# Intra- and Inter-Rater Reliability and Agreement of Ultrasound Imaging of Muscle Architecture and Patellar Tendon in Post-COVID-19 Patients Who Had Experienced Moderate or Severe COVID-19 Infection †

**DOI:** 10.3390/jcm11236934

**Published:** 2022-11-24

**Authors:** Leandro Gomes de Jesus Ferreira, Álvaro de Almeida Ventura, Isabella da Silva Almeida, Henrique Mansur, Nicolas Babault, João Luiz Quagliotti Durigan, Rita de Cássia Marqueti

**Affiliations:** 1 Laboratory of Muscle and Tendon Plasticity, Graduate Program in Rehabilitation Science, Faculdade de Ceilândia, Universidade de Brasília, Distrito Federal, Brasília 70910-900, Brazil; 2 Laboratory of Molecular Analysis, Graduate Program in Rehabilitation Science, Faculdade de Ceilândia, Universidade de Brasília, Distrito Federal, Brasília 70910-900, Brazil; 3 Department of Orthopaedics, Hospital Santa Helena—Rede D’or, Sao Paulo 03313-000, Brazil; 4 Centre d’Expertise de la Performance, INSERM U1093 CAPS, Sports Science Faculty, University of Burgundy, 21000 Dijon, France; 5 Graduate Program in Physical Education, Physical Education Department, Universidade de Brasília, Distrito Federal, Brasília 70910-900, Brazil

**Keywords:** inter-rater reliability, intra-rater reliability, muscle ultrasound, muscle thickness, ultrasound feasibility

## Abstract

COVID-19 is associated with musculoskeletal disorders. Ultrasound is a tool to assess muscle architecture and tendon measurements, offering an idea of the proportion of the consequences of the disease, since significant changes directly reflect the reduction in the ability to produce force and, consequently, in the functionality of the patient; however, its application in post-COVID-19 infection needs to be determined. We aimed to assess the intra- and inter-rater reliability of ultrasound measures of the architecture of the vastus lateralis (VL), rectus femoris (RF), vastus medialis (VM), gastrocnemius lateralis (GL), gastrocnemius medialis (GM), soleus (SO), and tibialis anterior (TA) muscles, as well as the patellar tendon (PT) cross-sectional area (CSA) in post-COVID-19 patients. An observational, prospective study with repeated measures was designed to evaluate 20 post-COVID-19 patients, who were measured for the pennation angle (θp), fascicular length (Lf), thickness, echogenicity of muscles, CSA and echogenicity of the PT. The intra-class correlation coefficient (ICC) and 95% limits of agreement were used. The intra-rater reliability presented high or very high correlations (ICC = 0.71–1.0) for most measures, except the θp of the TA, which was classified as moderate (ICC = 0.69). Observing the inter-rater reliability, all the evaluations of the PT, thickness and echogenicity of the muscles presented high or very high correlations. For the Lf, only the RF showed as low (ICC = 0.43), for the θp, RF (ICC = 0.68), GL (ICC = 0.70) and TA (ICC = 0.71) moderate and the SO (ICC = 0.40) low. The ultrasound reliability was acceptable for the muscle architecture, muscle and tendon echogenicity, and PT CSA, despite the low reliability for the Lf and θp of the RF and SO, respectively.

## 1. Introduction

In 2019–2020, the world witnessed a wave of distress and fear caused by the COVID-19 pandemic. By May 2022, over 500 million confirmed cases and over 6 million deaths had been reported globally [1]. Millions of people had been hospitalized with cardiovascular, nervous, muscular, and immune histological complications [2]. Some sequelae have been observed in COVID-19 patients, such as anorexia, weight loss, low albumin, fatigue, dyspnea, cough, anosmia, ageusia, and joint pain [3]. In addition, it seems that severe COVID-19 may be associated with systemic complications, such as cachexia [4], defined as “a complex metabolic syndrome associated with underlying illness and characterized by loss of muscle” by Morley et al. [4].

The loss of muscle strength and mass is the most common musculoskeletal complication related to the hospital length-of-stay in patients with moderate to severe COVID-19 [5]. The exact mechanism of muscle damage in COVID-19 patients, and the long-term muscle consequences in survivors, are not fully understood. However, the range of muscle mass and functional loss depends on several determinants, such as frailty, comorbidities, the level of the inflammatory response to COVID-19, anorexia, inadequate protein supply [6], and physical inactivity [6,7]. Immune-mediated mechanisms provide widely recognized explanations for the muscle impairment of SARS-CoV-2. This response is secondary to the cytokine storm and the awakening of the immune system [8]. A larger infiltration of immune cells was also observed in the skeletal muscles of patients who died from COVID-19 compared to patients who died from other critical illnesses [9], which may be related to the detriment of the musculoskeletal system in COVID-19 patients and could contribute to muscle weakness and fatigue. Andrade-Junior et al. [10] observed a reduction of 30.1% in the cross-sectional area (CSA) of the rectus femoris during ten days of hospitalization in COVID patients assessed by ultrasound imaging. To date, no studies have assessed the effects of COVID-19 on tendon remodeling, even though changes in tendons have been well described in situations of restriction of use [11,12]. This is particularly important, since patients who develop the most severe forms of the disease mostly end up hospitalized for days, contributing to related complications and several changes caused by muscle disuse [2].

Ultrasound imaging is one of the most common tools for evaluating muscle architecture and tendon properties in vivo [13,14]. Muscle architecture is defined by the organization of muscle fibers within a muscle affecting the force generation axis and is one of the main ways of measuring muscle function [15]. Briefly, the main outcomes are the pennation angle (the fiber angle relative to the force-generation axis, θp); fascicular length (the distance from the origin of the fascicle in the deep aponeurosis to the superficial aponeurosis, Lf), and muscle CSA, which presented good correlations with the muscle thickness (distance from deep aponeurosis to the superficial aponeurosis) [16]. Clinicians also record images and analyze tendon CSA and echogenicity (an index of tissue quality or fatty and fibrous infiltration) [17] to determine tissue quality in a non-invasive way. Previous studies have found that ultrasound imaging is reproducible for analyzing the properties of lower limb muscles and tendons [13,18,19]. However, to date, no studies have observed the intra- and inter-rater reliability of ultrasound imaging in post-COVID-19 patients. Clinicians are aware of the proportion of the consequences of the disease on these tissues, since significant changes in muscles and tendons of the lower limbs might directly reflect the reduction in the ability to produce force and, consequently, the functionality of COVID-19 patients.

As COVID-19 is a new and poorly studied disease, regardless of the comprehension related to musculoskeletal system dysfunction, ultrasound could be a useful tool to quickly assess the muscle and tendon properties of these patients outside and inside the hospital setting. Therefore, we aimed to examine the intra- and inter-rater reliability and agreement of the ultrasound measures of the architecture of the quadriceps femoris components (rectus femoris-RF, vastus lateralis-VL, and vastus medialis-VM), triceps surae components (gastrocnemius lateralis-GL, gastrocnemius medialis-GM, and soleus-SO) and tibialis anterior (TA), in addition to the CSA of the PT and echogenicity of all the muscles and tendons in post-COVID-19 patients. The hypothesis was that ultrasound is a reliable and reproducible tool to assess the muscles and tendons mentioned above in patients with moderate to severe COVID-19 when performed by the same rater and by different raters. Knowledge of the reliability of such measurements is essential in data capture standardization as well as in guiding clinical decisions.

## 2. Materials and Methods

### 2.1. Study Design

This is a prospective, observational blinded study with repeated measures to determine the intra- and inter-rater reliability of the measurements obtained by ultrasound images from the quadriceps femoris components, triceps surae, TA, and PT. We analyzed the muscle architecture (Lf, θp, thickness), the CSA of the PT, and the echogenicity of all the muscles and the PT, in post-COVID-19 participants. The research was conducted at the Laboratory of Clinical Physiology of the University of Brasilia, following the guidelines for reporting reliability and agreement studies (GRRAS) [20]. All the protocols were approved by the Human Research Ethics Board at the Faculty of Ceilândia (CAAE: 45043821.0.0000.8093) in accordance with the Declaration of Helsinki 1975. All the participants signed a consent form before the data collection. The current manuscript is part of a large observational study, and the full protocol is available at https://clinicaltrials.gov, identifier NCT04961255 (accessed on 9 November 2022).

### 2.2. Participants

A total of 20 participants were included in this study; of these, 12 patients had moderate COVID-19 (5 males and 7 females—mean (SD) age: 45.08 (12.58) years, body mass: 78.81 (23.60) kg, height: 1.68 (0.11) m), and 8 had severe COVID-19 (5 males and 3 females—age: 50.87 (9.50) years, body mass: 87.54 (6.74) kg, height: 1.66 (0.05) m) (Table 1 includes further information on the characteristics of the participants). The inclusion criteria for the study were patients aged 18–80 years who had experienced a moderate or severe COVID-19 infection according to Siddiqi and Mehra [2]. In addition, only patients who had been infected with COVID-19 within one year of study participation, considering the onset of symptoms/hospital discharge, were included. The exclusion criteria were body mass index (BMI) ≥ 35 kg/m^2^, reporting or diagnosis of swelling, skin damage, deformity or amputation in the regions to be examined, and behavioral problems that make it difficult to cooperate with the procedures of analysis.

### 2.3. Muscle and Tendon Ultrasound Imaging

The θp, Lf, thickness, CSA, and echogenicity were assessed using ultrasound (M-Turbo^®^, Sonosite, Bothwell, WA, USA) as described by Blazevich et al. [21]. The data were collected from the right limb (dominant limb) of all the participants. For the visualization and to obtain images of the RF, VL, VM, and TA, the participants were positioned in a supine position, with the lower limbs supported using a knee-bend to keep the muscle relaxed and reduce the fascicle curvature, in order to maximize the assessment reliability [21]. GL, GM, and SO images were obtained with patients in a prone position with the lower limbs fully extended and the ankle suspended off the stretcher.

**Figure 1:**
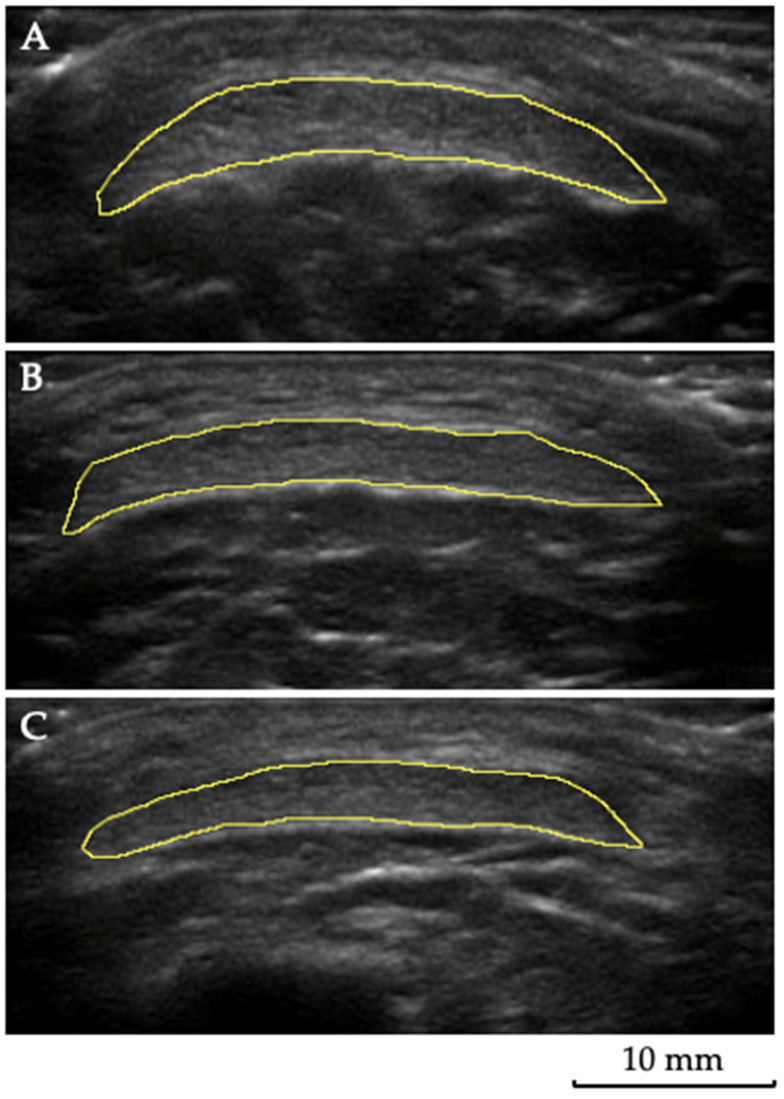
Tendon ultrasonography: a representative image of the patellar tendon ultrasound analysis. For this evaluation, the probe was placed at 25% (**A**), 50% (**B**), and 75%(**C**) of the length of the patellar tendon. The tendon contours were selected using the tracing technique to measure the cross-sectional area and echogenicity.

A water-soluble gel was applied to the probe to supply acoustic contact. The sequence of the muscle images obtained was randomized. The probe was positioned longitudinally on the tight calf to visualize the θp, Lf, and thickness. In contrast, the probe was placed in a transverse plane for the CSA of the PT and the echogenicity measurement. For the visualization of the RF, VL, and VM, the percentages of 50%, 60%, and 80%, respectively, of the distance between the medial aspect of the anterior superior iliac spine and the superior edge of the patella were considered [21]. The TA muscle was evaluated at the proximal 1/4 of the distance between the lower border of the patella and the lateral malleolus, with the transducer positioned on the anterolateral aspect of the leg [22], while the GL, GM, and SO muscles were evaluated, respectively, at 30, 30, and 50% of the distance from the popliteal crease to the lateral malleolus [23,24]. For ultrasound evaluation of the PT, the probe was placed at 25%, 50%, and 75% of the length of the PT [13,14] (Figure 1). The PT length was measured between the deep insertion in the patella and the deep insertion in the tibial tuberosity. The CSA measurement was performed considering the tendon contours.

**Figure 2:**
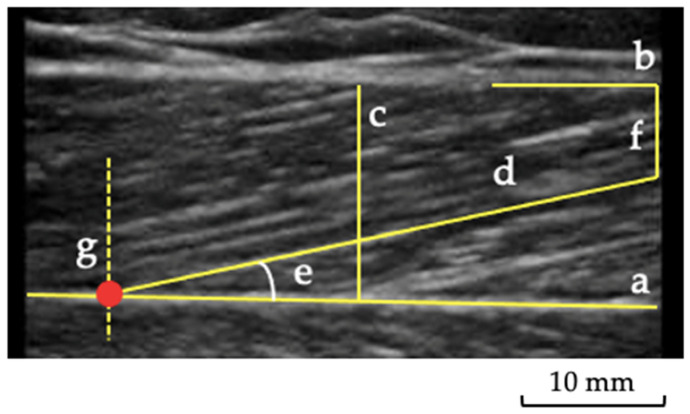
Muscle ultrasonography: a representative image of the gastrocnemius lateralis ultrasound analysis. (**a**) deep aponeurosis; (**b**) superficial aponeurosis; (**c**) muscle thickness; (**d**) fascicle length; (**e**) pennation angle; (**f**) distance between end of fascicle visualization and superficial aponeurosis; (**g**) lines indicating the cross-point between fascicle and deep aponeurosis.

Three images were obtained for each participant, and the average was considered for analysis. The θp was considered as the angulation of the fibers concerning the muscle’s line of action of force, and it was measured by assessing the angle formed between the muscle fiber and the deep aponeurosis [25] (Figure 2). The Lf was considered as the total length of the muscle fiber (Figure 2). Since the ultrasound probe was too small to visualize the whole fascicle from origin to insertion, corrections were made according to previous recommendations [21]. For the thickness, five lines were marked along the image, representing each muscle’s thickest area between the superficial and deep aponeurosis (Figure 2). Subsequently, the average of these five measurements was taken to obtain a more reliable value of muscle thickness [22]. For the muscle and tendon echogenicity, a region of interest was selected in each muscle, using the tracing technique, to include all the visible muscles in the ultrasonographic image without any bone or surrounding fascia [26]. A histogram was used to represent it on a grayscale, with values ranging from 0 to 255 (0: black/no wave reflection; 255: white/total wave reflection) [27,28], and the mean echogenicity of the region of interest was calculated and averaged over the three measurements per muscle and per each area of the PT.

To confirm that each researcher could locate and view the θp, Lf, thickness, echogenicity, and tendon characteristics, numerous practice sessions were performed using the portable ultrasound unit (M-Turbo^®^, Sonosite, Bothwell, WA, USA) in B-mode with a 7.5 Mhz linear transducer. Two raters performed the ultrasound measurements; both were physical therapists, and one had experience with ultrasound imaging and was considered the experienced rater (T1—ISA). The other, considered the novice rater, was trained for six months to perform the measurements (T2—LGJF). During the data collection, while one of the raters was collecting the images, the other was out of the laboratory. As an auxiliary, both the raters and the third physical therapist (T3) handled the ultrasound. All the images were analyzed with ImageJ software (National Institute of Health, Bethesda, MD, USA).

### 2.4. Statistical Analysis and Sample Size

The data were analyzed using SPSS 22.0 software (IBM Corporation, Armonk, NY, USA). The statistical level adopted was *p* < 0.05. The ultrasound data for each participant were obtained at the same time of day by the raters, who did not have access to each other’s measurements. A third researcher randomized the examiners’ evaluation order using the brown envelope method. Double and triple measures, calculated by the average of the records obtained in the two first and the three total records, were also used to form the five conditions of analysis: first (A1), second (A2), and third (A3) single trials recorded, as well as double (A1 + A2/2) and triple (A1 + A2 + A3/3) calculated measures. We chose the triple measure for the inter-rater reliability analysis because it represents the average of the three measurements made by each rater. For the intra-rater reliability analysis, we chose the three single trials recorded.

To interpret the magnitude of the correlation coefficients, the classification suggested by Mukaka [29] was adopted: 0.00 to 0.30, insignificant; 0.31 to 0.50, low; 0.51 to 0.70, moderate; 0.71 to 0.90, high; 0.91 to 1.00, very high. Subsequently, the Bland–Altman (BA) plot was used to verify absolute reliability. The Bland–Altman plot was examined as a visual representation of the intra- and inter-examiner agreement level and to assess the presence of a systematic error, considering a 95% confidence interval that constitutes the limits of agreement for the measures. The sample size was estimated a priori, using the guidelines from landmark studies [30] and considering the performance of three measurements for each analysis. For an α error < 0.05, power (1 − β) > 0.8, acceptable reliability (ρ0) of 0.70, and expected reliability (ρ1) of 0.90, a sample size of 20 participants was estimated, considering a 20% drop-out rate.

## 3. Results

### 3.1. Intra-Rater Reliability 

The majority of the measurements presented correlations classified as high or very high (ICC range from 0.71 to 1.0), except for the measure of the θp of the TA for the less experienced rater (ICC = 0.698), which was classified as moderate (Table 2). 

### 3.2. Inter-Rater Reliability

The measurements of the thickness and echogenicity of the muscles presented correlations classified as high or very high (ICC 0.71 to 1.0). In addition, analyzing the Lf, the correlations were classified as high (ICC 0.71 to 0.90), except for the RF, which showed low reliability (ICC = 0.435). For the θp, the VL, VM, and GM showed high reliability (ICC 0.71 to 0.90); however, the RF (ICC = 0.687), GL (ICC = 0.705), and TA (ICC = 0.712) showed moderate reliability, and the SO showed low reliability (ICC = 0. 403). All the positions of the PT had high or very high reliability for both the CSA and echogenicity (Table 3).

#### 3.2.1. Fascicular Length

**Figure 3:**
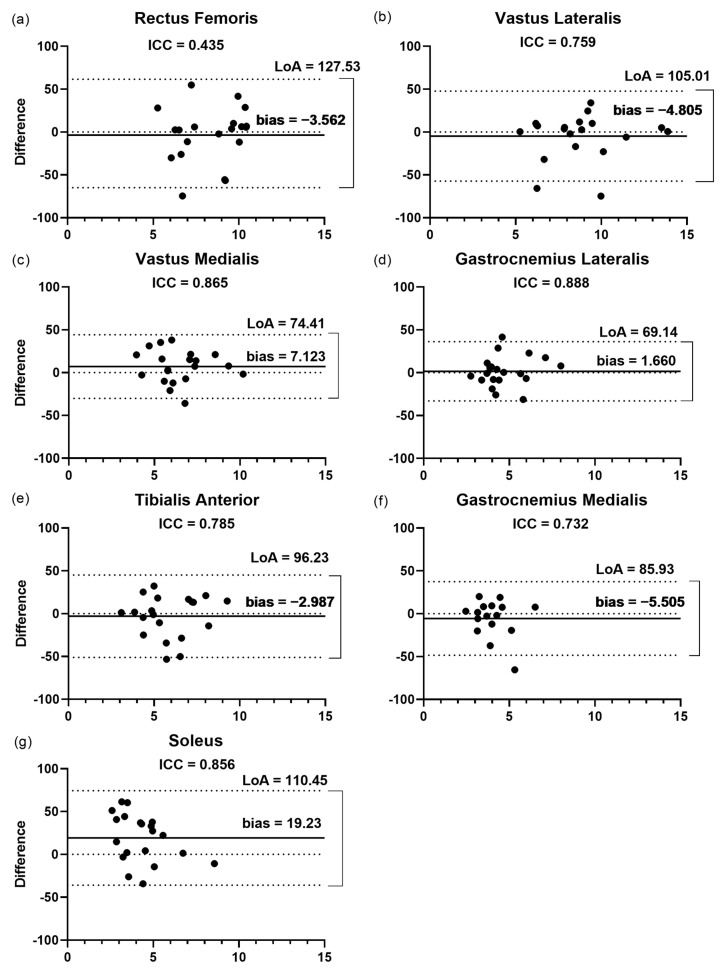
Bland–Altman plots displaying the differences (expressed as %) plotted against the average Lf recorded by the two raters for each muscle. (**a**–**g**) Inter-rater reliability of Lf measurement for (**a**) rectus femoris; (**b**) vastus lateralis; (**c**) vastus medialis; (**d**) gastrocnemius lateralis; (**e**) tibialis anterior; (**f**) gastrocnemius medialis; and (**g**) soleus. The lines show the zero, bias, and random error lines. Dashed lines represent the 95% limits of agreement (LoA). Lf, fascicular length; LoA, limits of agreement.

The Bland–Altman plots (Figure 3) showed that the LoA varied from 69.1% for the GL to 127.5% for the RF. The SO presented the highest bias (19.2%) among the muscles assessed, showing values ranging from 1.6 to 7.1. In agreement with the ICC results, the GL presented the highest correlation (ICC = 0.88) between the raters and the lowest bias (1.6%) and LoA (69.1%) (Figure 3d). In addition, the RF (Figure 3a) also showed a low correlation (ICC = 0.43) with the highest LoA and one of the lowest biases (−3.56%).

#### 3.2.2. Pennation Angle 

**Figure 4:**
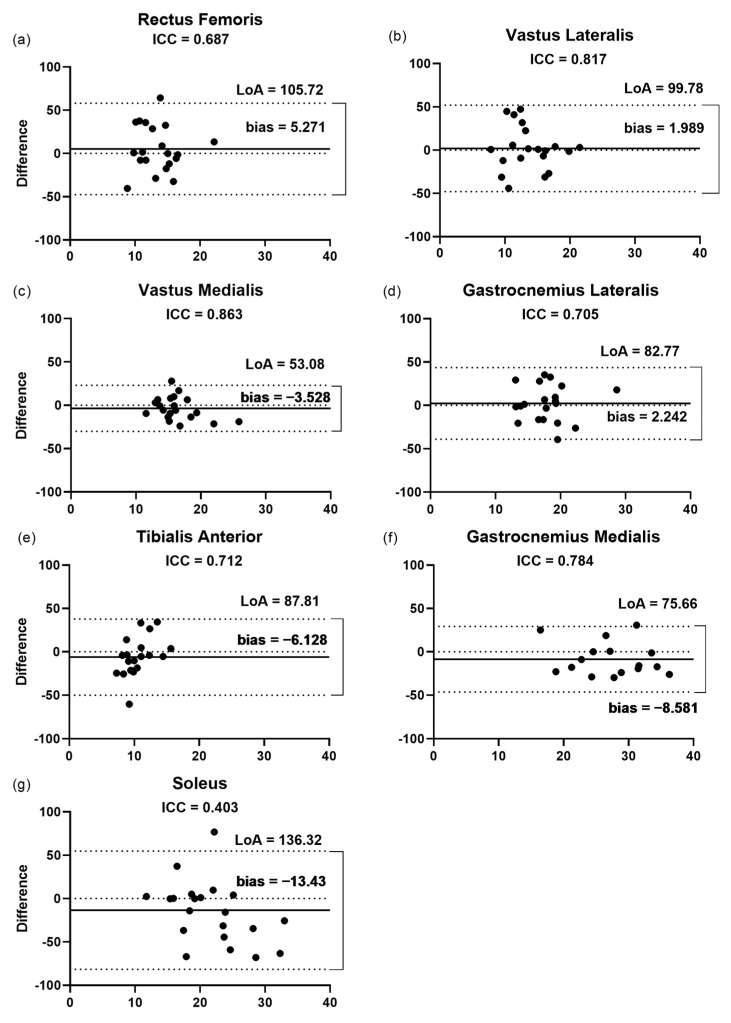
Bland–Altman plots displaying the differences (expressed as %) plotted against the average θp of each muscle recorded by the two raters. (**a**–**g**) Inter-rater reliability of θp measurement for (**a**) rectus femoris; (**b**) vastus lateralis; (**c**) vastus medialis; (**d**) gastrocnemius lateralis; (**e**) tibialis anterior; (**f**) gastrocnemius medialis; and (**g**) soleus. The lines show the zero, bias, and random error lines. Dashed lines represent the 95% limits of agreement (LoA). θp, pennation angle; LoA, limits of agreement.

The findings of the Bland–Altman plots (Figure 4) for the pennation angle measurements showed that the LoA varied from 53% for the VM to 136.3% for the SO. This was in agreement with the low correlation (ICC = 0.40) and the highest bias and LoA presented by the SO (13.43% and 136.32%, respectively, Figure 4g), in a range of 1.9 and 8.5% among the other muscles assessed. The lowest bias (1.9%) and LoA (99.7%) were observed for the VL (Figure 4b), even though the highest correlation (ICC = 0.86) was presented by the VM between the raters.

#### 3.2.3. Thickness

**Figure 5:**
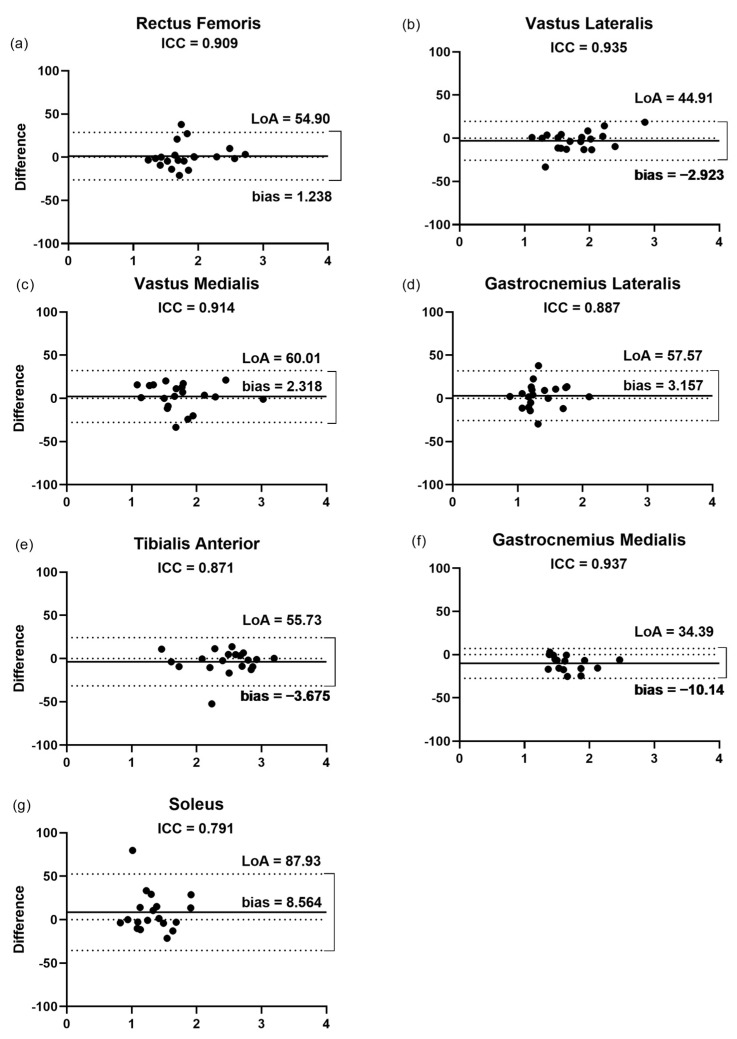
Bland–Altman plots displaying the differences (expressed as %) plotted against the average thickness recorded by the two raters for each muscle. (**a**–**g**) Inter-rater reliability of thickness measurement for (**a**) rectus femoris; (**b**) vastus lateralis; (**c**) vastus medialis; (**d**) gastrocnemius lateralis; (**e**) tibialis anterior; (**f**) gastrocnemius medialis; and (**g**) soleus. The lines show the zero, bias, and random error lines. Dashed lines represent the 95% limits of agreement (LoA). LoA, limits of agreement.

The findings of the Bland–Altman plots (Figure 5) for the thickness measurements showed that the LoA varied from 34.3% for the GM to 87.9% for the SO. In agreement, the GM muscle presented the highest correlation (ICC = 0.93) between the raters. This finding was not followed by the highest bias for the GM (10.1%), with a range of 1.2 to 8.5% among the other muscles assessed. The lowest bias (1.2%) was observed for the RF (Figure 5a).

#### 3.2.4. Echogenicity

**Figure 6:**
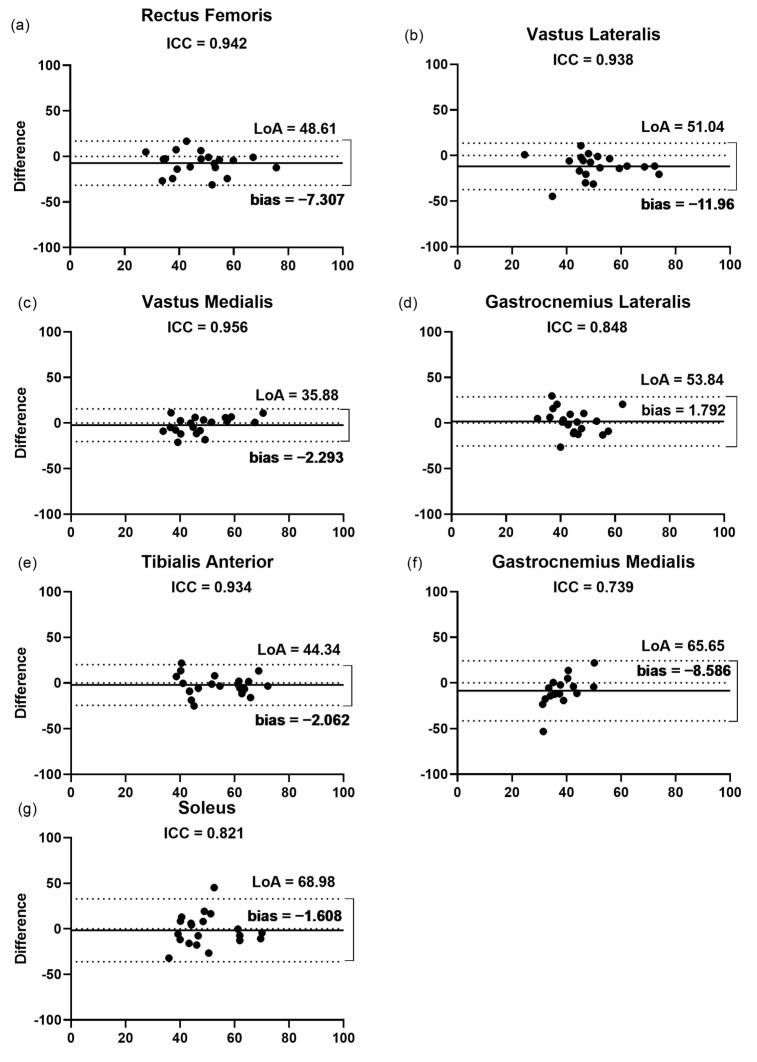
Bland–Altman plots displaying the differences (expressed as %) plotted against the average echogenicity recorded by the two raters for each muscle. (**a**–**g**) Inter-rater reliability of echogenicity measurement for (**a**) rectus femoris; (**b**) vastus lateralis; (**c**) vastus medialis; (**d**) gastrocnemius lateralis; (**e**) tibialis anterior; (**f**) gastrocnemius medialis; and (**g**) soleus. The lines show the zero, bias, and random error lines. Dashed lines represent the 95% limits of agreement (LoA). LoA, limits of agreement.

The findings of the Bland–Altman plots (Figure 6) for the echogenicity measurements showed that the LoA varied from 35.8% for the VM to 68.9% for the SO. The lowest bias (1.6%) and LoA (35.88%) were observed for the SO (Figure 6g) and VM (Figure 6c), respectively. Despite the highest bias for the VL (11.9%), with a range of 1.6 to 8.5 among the other muscles assessed, this muscle presented a very high correlation (ICC = 0.93).

#### 3.2.5. Cross-Sectional Area of the Patellar Tendon

**Figure 7:**
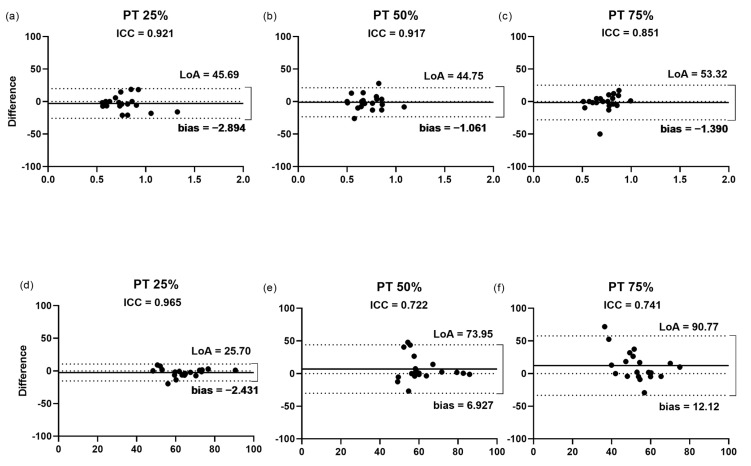
Bland–Altman plots displaying the differences (expressed as %) plotted against the average CSA and echogenicity recorded by the two raters for each PT position. Inter-rater reliability of CSA (**a**–**c**) and echogenicity (**d**–**f**) measurement for (**a**,**d**) 25%; (**b**,**e**) 50%; and (**c**,**f**) 75%. The lines show the zero, bias, and random error lines. Dashed lines represent the 95% limits of agreement (LoA). CSA, cross-sectional area; LoA, limits of agreement; PT, patellar tendon.

The findings of the Bland–Altman plots (Figure 7a–c) for the measurement of the CSA of the PT showed that the LoA varied from 44.7% at the position of 50% of the length to 53.3% at the position of 75%. This finding was not followed by the bias, which was higher at the position of 25% (2.8%), compared with the values of 1.3 and 1.0%, for the positions of 75 and 50%, respectively. The measurement of the CSA of all the positions presented high ICC with a low bias and LoA. However, despite the percentage of 50% showing the lowest bias and LoA (Figure 7b), the position with the highest correlation (ICC = 0.92) between the raters was at 25% of the tendon length.

#### 3.2.6. Echogenicity of the Patellar Tendon

The findings of the Bland–Altman plots (Figure 7d–f) for the echogenicity of the PT measurements showed that the LoA varied from 25.7% at the position of 25% of the length to 90.77% at the position of 75%. This finding was followed by the bias, which was highest at the position of 75% (12.1%), compared with the values of 2.4 and 6.9% for the positions of 25 and 50%, respectively. The lowest bias (2.4%) and LoA (25.7%) were observed for the measurement at the position of 25% (Figure 7d), which showed the highest correlation (ICC = 0.96) between the raters.

## 4. Discussion

To our knowledge, this is the first study to measure the intra- and inter-rater reliability and agreement of ultrasound measures of the architecture of the quadriceps femoris and triceps surae components and the TA, in addition to the CSA and echogenicity of the PT from post-COVID-19 patients. The results demonstrated that the measures presented a moderate to very high intra-rater reliability (ICC ranging from 0.51 to 1.00), for the majority of measurements, except for the RF Lf (ICC 0.43) and SO θp (ICC 0.40), which indicated low reliability. These findings are in agreement with the hypothesis that ultrasound is a reliable tool that allows an acceptable determination of the architecture of different lower limb muscles. Our results support the diagnosis and evaluation of musculoskeletal disorders of COVID-19 by clinicians and researchers and will also help to correctly interpret the studies presenting data on ultrasound imaging of muscle and tendons from COVID-19 patients. It is worth remembering that, although participants with different severities of COVID-19 were included in this study, the objective was to examine the reliability of ultrasound in the muscles of COVID-19 patients and not to compare the ultrasound measurements between the different groups. We are developing further studies to compare the musculoskeletal properties in participants exposed to COVID-19, “long COVID”, through ultrasound measurements. Huang et al. [31], demonstrated that fatigue or muscle weakness persisted in over 60% of patients six months after COVID-19 symptom onset, which substantially impacts the functionality and quality of life of COVID-19 survivors. However, the exact mechanism of musculoskeletal involvement in COVID-19 is not understood [32], and it is important to note that some points must be considered, such as the patient’s previous condition. In individuals with knee osteoarthritis, for example, joint tissue inflammation can contribute to the progression of synovial inflammation [33]. This can be an important factor in establishing the sequelae and/or changes in the musculotendinous architecture of a COVID-19 infection. Therefore, due to the global impact of the COVID-19 pandemic, diagnostic tools are needed to assess the musculotendinous quality of these patients. One of these tools is ultrasound, a non-invasive, easy-to-use, and portable device that enables quick access to a visualization of musculoskeletal imaging properties [34,35].

Concerning the muscle architecture, our results are in partial agreement with those presented in a previous review [36]. While the authors reported good reliability for the ultrasound measures of the relaxed muscles of the lower limb, we found low reliability for the RF Lf and SO θp. In addition, the majority of studies reported moderate to high-reliability estimates for the θp and Lf of the VL [19,37,38,39], RF [37,39], TA [38], GM [40,41,42], and GL [42]; while the studies exploring these measurements using ultrasound in SO [43] and VM [44] did not report reliability estimates. This fact makes it difficult to compare our reliability findings for the SO and VM with the previous studies.

An ultrasound assessment may be a convenient, non-invasive tool to evaluate muscle wasting and quality, especially when assessing muscle thickness and echogenicity [45,46]. Here, our results demonstrated a high or very high intra- and inter-rater reliability for these two measurements. These results agree with Karapinar et al. [17], who investigated the reliability of measuring the echogenicity features of the quadriceps muscle in patients with knee osteoarthritis. In addition, Pardo et al. [47] observed an ICC of 0.83 for the thickness of quadriceps femoris inter-rater reliability in critically ill patients, and May et al. [42] showed excellent measurements (ICC 0.81 to 0.88) for the thickness of the GL and GM in a healthy population.

In addition to muscle changes, prolonged disuse affects tendon properties, such as by reducing tissue stiffness, CSA, and tendon thickness [11,48]. Therefore, using ultrasound as a tendinous assessment tool seems to represent a good possibility. Previous studies demonstrated acceptable reliability for the ultrasound measures of the CSA of the PT in healthy individuals, expressing ICCs ranging from 0.87–0.98 [49] and 0.89 to 0.98 for critically ill patients [13]. Concerning the PT, only one study investigated the reliability of three positions (25%, 50%, and 75%) in critically ill patients [13]. The magnitude of the reliability of tendon CSA and echogenicity was classified as “almost complete”, irrespective of the rater, confirming the results obtained in the present study.

Although the ultrasound reliability of the muscle architecture, muscle thickness, muscle and tendon echogenicity, and PT CSA images taken by the independent raters was acceptable, we observed low inter-rater reliability for the RF Lf and SO θp. These results could be related to more pronounced changes in the muscular architecture of these muscles, resulting from the infection process, which may require greater caution during ultrasound analysis. The RF is an important knee extensor muscle and also has an action in hip flexion, and the SO is a deeper muscle that is difficult to visualize because it is below the gastrocnemius muscle [50,51]. It is possible that these anatomical characteristics also affect the analysis. In addition, this could be explained by a common error that can appear during ultrasound measurements: the inconsistent alignment of the probe, as previously reported [52]. Moreover, a muscle is a series of interconnected planes. Some authors have indicated that an optimal orientation is such that the fascicles can be visualized along their length from the superficial to the deep aponeuroses, where the plane of the ultrasound is parallel to the fascicles [24]. Otherwise, the fascicle length would be overestimated, and the fascicle angle would be underestimated, which suggests that probe rotation can affect the measures of Lf and θp during an ultrasound analysis [52].

The importance of applying ICC values accompanied by the Bland–Altman method for reliability studies has been shown [53,54]. We observed an agreement between both assessments, with high reliability accompanied by low bias and LoA, and low ICC values for measures with a high bias and LoA. However, this pattern was not observed for some measures. For example, the θp and thickness of the GM showed a high bias (8.5 and 10.1), despite a moderate or high ICC (0.78 and 0.93, respectively). A similar result occurred for the measures of the echogenicity of the VL and PT at 75%. These results may occur due to the difficulty of taking these measurements using the ultrasound technique, or because of a higher standard error measurement of the novice rater. The previous test-retest studies ratified this possible learning effect and reported low inter-rater reliability related to the different levels of experience between the raters [55]. 

The main limitation of this study was the inclusion of post-COVID-19 patients who had undergone a long period of disuse and had more pronounced musculoskeletal repercussions of the disease, which may make the analysis more challenging. In addition, the number of participants is small for adequate powering, but the sample size and statistical analysis were adequate for the preliminary formulation of a hypothesis yet to be tested in a larger project. Therefore, this group of participants with different degrees of disease severity and further musculoskeletal sequelae may influence the image acquisition. In addition, there is a lack of studies that measured the muscle and tendon ultrasound imaging of the quadriceps femoris, triceps surae components, and TA in post-COVID-19 participants. Thus, it was difficult to compare all of our results with previous studies or extrapolate our results to other populations, such as healthy individuals. Collectively, this study is the initial step to disseminating the use of this ultrasound imaging tool to evaluate muscle and tendon imaging in post-COVID-19 patients. Ultrasound imaging could be fundamental to understanding the symptoms of long COVID-19 in order to develop a treatment strategy to more quickly improve the quality of life of these patients. Therefore, future studies are needed to detect the longitudinal changes in different muscles and tendons in post-COVID-19 patients, especially those that closely analyze joint structures, such as the knee retinaculum and patellofemoral ligaments, since these structures are extremely important for patellar stabilization [56] and may undergo changes in their architecture and thus affect the functionality.

## 5. Conclusions

The ultrasound imaging reliability taken by the independent raters was acceptable and mostly not influenced by the rater’s experience for the muscle architecture, muscle thickness, muscle, tendon echogenicity, and PT CSA, despite the low reliability for the RF Lf and SO θp. In this study, ICC provided the reliability index that reflects both the degrees of correlation and agreement between the measurements, and that ultrasound is an excellent, reproducible method to evaluate the muscle architecture in COVID-19 patients.

## Figures and Tables

**Table 1:** Characteristics of the participants.

	Total (*n* = 20)
Age (years)	46.89 (11.64)
Sex	
Male	10 (50%)
Female	10 (50%)
Body mass (Kg)	82.00 (19.41)
Height (m)	1.67 (0.09)
BMI (kg/m^2^)	28.92 (5.02)
Education	
College or Higher	18 (90%)
Middle school or lower	2 (10%)
Comorbidities	
Hypertension	8 (40%)
Diabetes	4 (20%)
Cardiovascular disease	2 (10%)
Cerebrovascular disease	1 (5%)
Hyperlipidemia	6 (30%)
Depression	1 (5%)
Panic Syndrome	2 (10%)
Anxiety	2 (10%)
Asthma	3 (15%)
Tumors	1 (5%)
Time from symptom onset to analysis (days)	114.25 (89.91)
Hospitalization—yes/no	8/12
Length of hospital stay (days)	20.87 (20.97)
Length of ICU stay (days)	14.37 (18.76)
Vaccine	10 (50%)
Smoking history	3 (15%)

Data are reported as mean (SD) or frequency (%). BMI = body mass index; ICU = intensive care unit. Length of hospital stay and length of ICU stay were calculated considering patients who were hospitalized.

**Table 2:** Intra-rater reliability and 95% CI of the muscle and tendon architecture and echogenicity from the sample (*n* = 20) of Raters 1 and 2.

		Rater 1 (T1)		Rater 2 (T2)	
Muscles		ICC	95% CI	ICC	95% CI
RF	Lf	0.89	0.77–0.95	0.88	0.75–0.95
	θp	0.90	0.79–0.95	0.92	0.84–0.96
	Thickness	0.99	0.98–0.99	0.99	0.99–0.99
	Echogenicity	0.98	0.97–0.99	0.99	0.99–0.99
VL	Lf	0.89	0.78–0.95	0.92	0.83–0.96
	θp	0.89	0.78–0.95	0.91	0.81–0.96
	Thickness	0.99	0.98–0.99	0.99	0.99–0.99
	Echogenicity	0.98	0.96–0.99	0.99	0.99–0.99
VM	Lf	0.80	0.58–0.91	0.95	0.90–0.98
	θp	0.71	0.39–0.87	0.92	0.85–0.97
	Thickness	0.99	0.99–0.99	0.96	0.99–0.99
	Echogenicity	0.99	0.98–0.99	0.99	0.98–0.99
TA	Lf	0.93	0.85–0.97	0.91	0.82–0.96
	θp	0.87	0.73–0.94	0.69	0.37–0.87
	Thickness	0.99	0.97–0.99	0.99	0.98–0.99
	Echogenicity	0.98	0.97–0.99	0.98	0.96–0.99
GL	Lf	0.96	0.91–0.98	0.94	0.87–0.97
	θp	0.94	0.87–0.97	0.95	0.90–0.98
	Thickness	0.99	0.98–0.99	0.97	0.95–0.99
	Echogenicity	0.97	0.93–0.98	0.98	0.96–0.99
GM	Lf	0.94	0.88–0.98	0.97	0.94–0.99
	θp	0.94	0.87–0.97	0.96	0.92–0.98
	Thickness	0.98	0.96–0.99	0.99	0.99–0.99
	Echogenicity	0.99	0.97–0.99	0.95	0.89–0.98
SO	Lf	0.92	0.84–0.96	0.97	0.95–0.99
	θp	0.87	0.74–0.94	0.97	0.94–0.99
	Thickness	0.98	0.96–0.99	0.99	0.98–0.99
	Echogenicity	0.99	0.97–0.99	0.99	0.99–0.99
PT					
25%	CSA	0.97	0.95–0.99	0.97	0.95–0.99
	Echogenicity	0.95	0.89–0.97	0.97	0.94–0.98
50%	CSA	0.96	0.91–0.98	0.97	0.95–0.99
	Echogenicity	0.97	0.95–0.99	0.98	0.97–0.99
75%	CSA	0.94	0.88–0.97	0.96	0.93–0.98
	Echogenicity	0.92	0.84–0.96	0.98	0.97–0.99

ICC, intra-class correlation coefficient; CI, confidence interval; RF, rectus femoris; VL, vastus lateralis; VM, vastus medialis; TA, tibialis anterior; GL, gastrocnemius lateralis; GM, gastrocnemius medialis; SO, soleus; Lf, fascicle length; θp, pennation angle; PT, patellar tendon; CSA, cross-sectional area.

**Table 3:** Inter-rater reliability and 95% CI of the muscle and tendon architecture and echogenicity obtained from the sample (*n* = 20) of Raters 1 and 2.

	Lf (ICC–95% CI)		θp		Thickness (ICC–95% CI)		Echogenicity	
			(ICC–95% CI)				(ICC–95% CI)	
RF	0.43	0.42–0.77	0.68	0.21–0.87	0.90	0.77–0.96	0.94	0.85–0.97
VL	0.75	0.39–0.90	0.81	0.53–0.92	0.93	0.83–0.97	0.93	0.84–0.97
VM	0.86	0.65–0.94	0.86	0.65–0.94	0.91	0.78–0.96	0.95	0.88–0.98
TA	0.78	0.45–0.91	0.71	0.27–0.88	0.87	0.67–0.94	0.93	0.83–0.97
GL	0.88	0.71–0.95	0.70	0.25–0.88	0.88	0.71–0.95	0.84	0.61–0.94
GM	0.73	0.23–0.90	0.78	0.32–0.92	0.93	0.81–0.97	0.73	0.25–0.90
SO	0.85	0.63–0.94	0.40	0.50–0.76	0.79	0.47–0.91	0.82	0.54–0.92
	CSA (ICC–95% CI)				Echogenicity (ICC–95% CI)			
PT 25%	0.92		0.79–0.96		0.96		0.91–0.98	
PT 50%	0.91		0.79–0.96		0.72		0.29–0.89	
PT 75%	0.85		0.62–0.94		0.74		0.34–0.89	

ICC, intra-class correlation coefficient; CI, confidence interval; RF, rectus femoris; VL, vastus lateralis; VM, vastus medialis; TA, tibialis anterior; GL, gastrocnemius lateralis; GM, gastrocnemius medialis; SO, soleus; Lf, fascicle length; θp, pennation angle; PT, patellar tendon; CSA, cross-sectional area.

## Data Availability

Not applicable.
